# Characteristics of ill-demarcated small pancreatic ductal adenocarcinoma on MRI: a multi-institutional retrospective cohort study

**DOI:** 10.1007/s11604-026-02003-8

**Published:** 2026-05-20

**Authors:** Shogo Takeuchi, Yoshihiko Fukukura, Takeshi Fukunaga, Keitaro Sofue, Dai Inoue, Atsushi Urase, Katsuhiro Sano, Yasunari Fujinaga, Nobuhiro Fujita, Yoshiki Asayama, Masamitsu Hatakenaka, Yoshifumi Noda, Satoshi Goshima

**Affiliations:** 1 Department of Radiology, Kawasaki Medical School, 577 Matsushima, Kurashiki City, Okayama 701-0192 Japan; 2 Department of Radiology, Kobe University Graduate School of Medicine, 7-5-2 Kusunoki-cho, Chuo-ku, Kobe, Hyogo 650-0017 Japan; 3 Department of Radiology, Kanazawa University Graduate School of Medical Sciences, 13-1 Takara-Machi, Kanazawa, Ishikawa 920-8640 Japan; 4 Department of Radiology, Kindai University Hospital, 1-14-1 Miharadai, Minami-ku, Sakai, Osaka 590-0197 Japan; 5 Department of Radiology, Juntendo University Graduate School of Medicine, 2-1-1 Hongo, Bunkyo-ku, Tokyo 113-8421 Japan; 6 Department of Radiology, Shinshu University School of Medicine, 3 Chome-1-1 Asahi, Matsumoto, Nagano 390-8621 Japan; 7 Department of Clinical Radiology, Graduate School of Medical Sciences, Kyushu University, 3-1-1 Maidashi, Higashi-ku, Fukuoka, 812-8582 Japan; 8 Department of Radiology, Oita University Faculty of Medicine, 1 Chome-1 Hasamamachi Idaigaoka, Yufu, Oita, Oita 879-5593 Japan; 9 Department of Diagnostic Radiology, School of Medicine, Sapporo Medical University, 17 Chome Minami 1 Jonishi, Chuo Ward, Sapporo, Hokkaido 060-8556 Japan; 10 Department of Radiology, Gifu University, 1-1 Yanagido, Gifu, 501-1194 Japan; 11 Department of Radiology, Hamamatsu University School of Medicine, 1-20-1, Handayama, Chuo-ku, Hamamatsu City, Shizuoka 431-3192 Japan

**Keywords:** Pancreatic neoplasms, Diagnostic imaging, Magnetic resonance imaging, Tumor delineation, Secondary signs, Pancreatic ducts

## Abstract

**Purpose:**

To investigate the frequency of visually ill-demarcated small pancreatic ductal adenocarcinomas (PDACs) and compare their clinicopathologic and MRI characteristics with those of well-demarcated tumors.

**Materials and methods:**

This multicenter retrospective study enrolled 210 patients with surgically confirmed small PDACs (≤ 20 mm) who underwent preoperative unenhanced MRI. Clinical and pathological data were collected. Two radiologists independently evaluated the following MRI features: (a) tumor delineation (well-demarcated or ill-demarcated), (b) tumor signal intensity, and (c) secondary signs, including distal parenchymal signal intensity, parenchymal atrophy, main pancreatic duct (MPD) stenosis and dilatation, and small retention cyst. Clinicopathologic features and MRI findings were compared between well-demarcated and ill-demarcated PDACs using the Mann–Whitney and Fisher’s exact tests. Multivariate logistic regression analysis was performed to identify independent factors associated with ill-demarcated PDAC.

**Results:**

Visually ill-demarcated tumors accounted for 46.7% of small PDACs. Compared with well-demarcated tumors, ill-demarcated tumors were associated with less frequent elevation of tumor marker levels (*P* = 0.001) and smaller tumor size (*P* < 0.001). Abnormal distal parenchymal signal intensity was observed more frequently on T1-weighted imaging than on diffusion-weighted imaging (*P* = 0.012) or T2-weighted imaging (*P* < 0.001), and was significantly more frequent in ill-demarcated tumors across all MRI sequences (all *P* < 0.05). MPD stenosis and dilatation on T2-weighted imaging were more frequent in ill-demarcated tumors (both *P* < 0.001). Ill-demarcated tumors were also more frequently associated with at least one secondary sign (*P* = 0.002). On multivariable analysis, smaller tumor size (*P* = 0.002) and the presence of MPD dilatation (*P* = 0.002) were independently associated with ill-demarcated PDAC.

**Conclusion:**

Nearly half of the small PDACs were visually ill-demarcated on MRI. Therefore, when a focal tumor is not clearly visible, careful assessment of secondary MRI findings, particularly distal parenchymal signal abnormalities on T1-weighted imaging and MPD dilatation on T2-weighted imaging, may aid in the diagnosis.

**Supplementary Information:**

The online version contains supplementary material available at https://doi.org/10.1007/s11604-026-02003-8.

## Introduction

Pancreatic ductal adenocarcinoma (PDAC) is currently the third most common cause of cancer-related mortality in the United States [1]. While the incidence of PDACs has increased in recent decades, the mechanisms remain unelucidated. The prognosis for patients with PDAC is poor, with a 5-year survival rate of approximately 13% [1], despite advancements in imaging and treatment. The only potentially effective treatment is surgical resection with negative margins. However, > 80% of patients are diagnosed at advanced, unresectable stages [2–6]. In contrast, small PDACs have a substantially better prognosis, with a 5-year survival rate of 80.4% for tumors ≤ 10 mm in diameter and 50.0% for those 10–20 mm in diameter [7]. Therefore, improving survival outcomes depends largely on earlier detection of PDACs, underscoring the need for effective, noninvasive diagnostic strategies.

Magnetic resonance imaging (MRI) has increasingly been used for the diagnosis of pancreatic diseases, providing high-contrast resolution that enables precise tumor demarcation without the need for contrast material [8–13]. However, MRI often fails to clearly delineate PDACs owing to tumor-associated pancreatitis [9–11]. Awareness of visually ill-demarcated PDACs is clinically important, as an obscured lesion on MRI may result in missed or delayed diagnosis. Nevertheless, visually ill-demarcated PDACs on MRI remain poorly understood. To our knowledge, the clinicopathologic and MRI characteristics of ill-demarcated PDACs have not yet been comprehensively evaluated.

Therefore, this study investigated the frequency of visually ill-demarcated small PDACs and compared their clinicopathologic and MRI characteristics with those of well-demarcated tumors.

## Materials and methods

### Patient cohort

This multi-institutional retrospective study was approved by the institutional review boards of 12 participating centers, who waived the requirement for informed consent. Consecutive eligible patients with histologically confirmed small (≤ 20 mm in diameter) PDAC who underwent preoperative MRI and surgical resection between January 2007 and December 2024 were retrospectively enrolled.

**Fig. 1:**
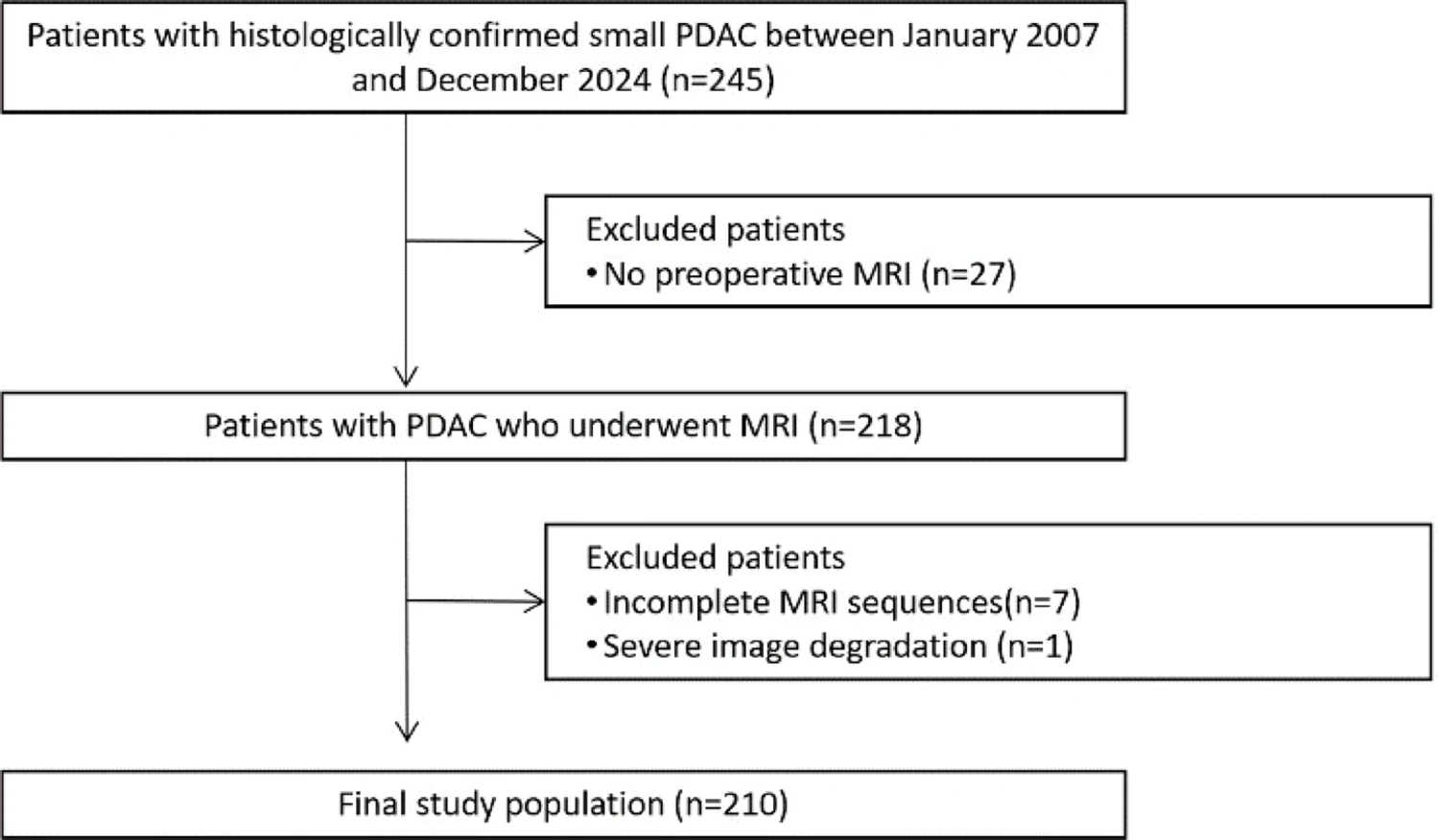
Flow diagram illustrating the patient selection process based on the inclusion and exclusion criteria

The inclusion criteria were the following: (a) pathologically confirmed PDAC with a maximal diameter ≤ 20 mm on surgical specimens, excluding PDAC associated with intraductal papillary mucinous neoplasm; and (b) availability of preoperative MRI performed within 3 months before surgery. The exclusion criteria were (i) any treatment for PDAC prior to MRI and surgical resection, (ii) incomplete MRI sequences (T1-, T2-, or diffusion-weighted imaging [DWI]), and (iii) severe image degradation due to motion or susceptibility artifacts (Fig. 1).

### Clinical data collection

Patient clinical data were extracted from the medical records, including sex, age, mode of tumor detection (incidental detection during evaluation of non-PDAC conditions, medical check-up, or symptoms), and preoperative serum tumor markers, namely carcinoembryonic antigen (CEA) and carbohydrate antigen 19 − 9 (CA19-9). Data retrieved from pathology reports included tumor size and location (head or body to tail), histological tumor grade, and TNM stage according to the 9th edition of the Union for International Cancer Control (UICC) TNM classification.

### Imaging technique

Pancreatic MRI was performed with a 1.5 T system (*n* = 84; Intera Achieva or Ingenia, Philips Medical Systems, Best, the Netherlands; Signa HDx, Signa Excite, or Signa Explorer or Optima MR360, GE Healthcare, Waukesha, WI, USA; Magnetom Avanto or Magnetom Essenza, Siemens Healthcare, Erlangen, Germany; and MRT-200SP5, Excelart Vantage, Vantage Titan, Vantage Orian, Canon Medical Systems, Tochigi, Japan) or a 3.0 T system (*n* = 126; Ingenia or Achieva, Philips Medical Systems; Magnetom Prisma, Trio, Vida, Spectra, or Skyra, Siemens Healthcare; Discovery MR750w or Signa Excite, GE Healthcare;. Vantage Titan, Centurian, or Galan, Canon Medical Systems), equipped with an excitation body coil and a phased array coil for signal reception.

The MRI acquisition parameters varied across the institutions, all examinations included standard pancreatic MRI sequences, enabling consistent evaluation of tumor delineation, tumor signal intensity, and secondary signs. T1-weighted images were obtained with two-dimensional (2D) or three-dimensional (3D) dual gradient-echo T1-weighted imaging in all patients and fat-suppressed T1-weighted imaging in 207 patients. In 2D imaging, TR ranged from 130 to 300 ms, with dual-echo TE values of 2.3–2.4 and 4.2–4.5 ms at 1.5 T or 1.1–1.2 and 2.3–2.4 ms at 3.0 T. In 3D imaging, TR ranged from 2.0 to 5.0 ms with the same dual-echo TE combinations. Slice thickness ranged from 4 to 8 mm for 2D imaging and 1.5–5 mm for 3D imaging. All patients underwent T2-weighted imaging using either breath-hold single-shot fast spin-echo (SSFSE) (TR/TE = ∞/75–150 ms) or respiratory-triggered fast spin-echo (FSE) (TR/TE = respiratory interval/65–120 ms), with a slice thickness of 4–8 mm; in addition, fat-suppressed T2-weighted imaging was performed in 193 patients. Magnetic resonance cholangiopancreatography (MRCP) was performed in 186 patients, utilizing either 2D single-shot fast spin-echo (TR/TE = ∞/95–1,100 ms; slice thickness, 3–50 mm) or 3D respiratory-triggered fast spin-echo (TR/TE = respiratory interval/65–800 ms; slice thickness, 1–2.5 mm). Furthermore, DWI was acquired in all patients using single-shot echo-planar imaging with b-values of 800 s/mm² (*n* = 92) or 1,000 s/mm² (*n* = 118) and a slice thickness of 4–8 mm. Detailed MRI acquisition parameters for each protocol are provided in Supplementary Table 1.

## Image analysis

Two board-certified radiologists (Y.F. and T.F., with 23 and 10 years of experience in abdominal imaging, respectively) independently reviewed the MR images, including T1-weighted, T2-weighted, MRCP, and DW sequences. The readers were blinded to all clinical data and to findings from other imaging modalities, except for knowledge of the PDAC diagnosis and the absence of other pancreatic tumors. The following morphologic features were assessed on T1-weighted imaging, T2-weighted imaging, MRCP, or DWI: (a) tumor demarcation; (b) tumor signal intensity; and (c) secondary signs, including distal parenchymal signal intensity; parenchymal atrophy (on T1-weighted images only); and main pancreatic duct (MPD) stenosis and dilatation, common bile duct (CBD) stenosis with upstream dilatation, or a small retention cyst adjacent to the tumor (on T2-weighted images and MRCP).

Tumor demarcation was classified as well-demarcated if the tumor was clearly and circumferentially distinguishable from the surrounding pancreatic parenchyma on at least one MRI sequence based on signal intensity differences. Tumors that were only partially distinguishable or were indistinguishable on all sequences were classified as ill-demarcated. Tumor signal intensity was evaluated relative to the surrounding pancreatic parenchyma on T1-weighted imaging, T2-weighted imaging, and DWI and categorized as hypointensity, isointensity, or hyperintensity. Distal parenchymal signal intensity was assessed in the pancreatic parenchyma distal to the tumor on T1-weighted imaging, T2-weighted imaging, and DWI and was categorized as hypointensity, isointensity, or hyperintensity. Parenchymal atrophy was categorized as focal or upstream to the tumor. Parenchymal atrophy was defined as a maximum anteroposterior pancreatic thickness < 10 mm [14–17]. Focal parenchymal atrophy was defined as a parenchymal indentation > 5 mm in the anteroposterior direction with a longitudinal extent > 10 mm along the upstream–downstream axis of the pancreas compared with the adjacent upstream and downstream parenchyma [18]. Upstream parenchymal atrophy, in cases with MPD dilatation, was defined as an MPD–to–total pancreatic parenchymal width ratio < 0.50 [15–19]. MPD stenosis was defined as luminal narrowing of the MPD, with or without upstream ductal dilatation. MPD dilatation was defined as an MPD diameter > 3 mm [14, 15, 19]. CBD stenosis was defined as luminal narrowing of the CBD with upstream bile duct dilatation > 8 mm attributable to the tumor [20]. A small retention cyst was defined as a round cystic lesion ≤ 5 mm in diameter adjacent to the tumor, without surrounding ducts or tubular structures communicating with the MPD [12].

Nonconcordant interpretations between the two readers were resolved by a consensus in consultation with a third board-certified radiologist (K.S., with 18 years of experience in abdominal imaging).

### Statistical analysis

All the data were analyzed statistically, using MedCalc Statistical Software (version 11.4; MedCalc Software Ltd., Mariakerke, Belgium). Continuous variables were expressed as mean ± standard deviation and categorical variables as number (percentage). Cohen’s kappa (κ) or weighted kappa statistic was used to evaluate interobserver agreements for MRI features, with an agreement defined as follows: κ = 0.00–0.20, slight; κ = 0.21–0.40, fair; κ = 0.41–0.60, moderate; κ = 0.61–0.80, substantial; and κ = 0.81–1.00, almost perfect agreement [21].

Cochran’s Q test was used to compare tumor demarcation, tumor signal intensity, and distal parenchymal signal intensity among the three MRI sequences. When a significant difference was identified, post hoc pairwise comparisons were performed using McNemar tests with Bonferroni correction. The Mann–Whitney U test and Fisher’s exact test were used to compare clinicopathological characteristics, including the interval between MRI and surgery, and MRI features between well-demarcated and ill-demarcated small PDACs. Univariate logistic regression analysis was performed to evaluate the associations of clinicopathological features and MRI secondary signs (parenchymal atrophy, MPD stenosis and dilatation, CBD stenosis, and small retention cysts) with ill-demarcated small PDAC, using well-demarcated tumors as the reference category. Variables with *P* < 0.10 in the univariate analysis were subsequently included in a multivariate logistic regression model to identify independent factors. To evaluate the potential influence of interinstitutional heterogeneity in MRI acquisition parameters, additional exploratory subgroup analyses were performed using Fisher’s exact test to compare the distribution of well- and ill-demarcated tumors according to T1-weighted imaging technique (2D vs. 3D), T2-weighted imaging technique (SSFSE vs. FSE), and DWI b-value (800 vs. 1000 s/mm²). A two-sided *P*-value < 0.05 was considered statistically significant.

## Results

### Clinicopathological characteristics (Table 1)

Of 245 patients with histologically confirmed small PDAC, 218 met the inclusion criteria. However, 8 were excluded, yielding a final cohort of 210 patients (101 male, 109 female; mean age, 70.6 years; range, 39–89 years) (Fig. 1). In total, 46.7% (*n* = 98) of tumors were classified as visually ill-demarcated tumors. The tumor groups did not differ significantly in sex (*P* = 0.213), age (*P* = 0.230), or mode of tumor detection (*P* = 0.293). For the entire cohort, the mean interval between MRI and surgery was 0.80 ± 0.64 months (range, 0–3.0 months). The interval did not differ significantly between well-demarcated and ill-demarcated tumors (*P* = 0.399). Elevation of CA19-9 levels was less frequent in ill-demarcated tumors (*P* = 0.001), whereas CEA levels were not significantly different between the groups (*P* = 0.272).

Ill-demarcated tumors were significantly smaller in size (*P* < 0.001) and were more frequently located in the body to tail of the pancreas (*P* = 0.036) compared with well-demarcated tumors. No significant differences in histological tumor grade (*P* = 0.408) or TNM stage (*P* = 0.287) were observed between the groups.

**Table 1:** Patient and tumor characteristics

	Well-demarcated PDAC		Ill-demarcated PDAC		*P*-value
	(*n* = 112)		(*n* = 98)		
*Clinical characteristics*					
Sex (male/female)	49/63		52/46		0.213
Age (years)*	71.3 ± 9.6	(39–89)	69.9 ± 8.8	(47–86)	0.230
*Opportunities for detecting PDAC*					0.293
Incidental detection during evaluation of non-PDAC conditions	45.5%	(50/110)	53.6%	(52/97)	
Medical check-up	27.3%	(30/110)	18.6%	(18/97)	
Symptoms	27.3%	(30/110)	27.8%	(27/97)	
*Interval between MRI and surgery (months)*	0.73 ± 0.55	(0.0–2.7)	0.87 ± 0.74	(0.0–3.0)	0.399
*Tumor markers*					
CEA (> 5.68 ng/mL)	13.5%	(15/111)	8.2%	(8/97)	0.272
CA19-9 (> 37.0 U/mL)	51.8%	(58/112)	28.9%	(28/97)	0.001
*Tumor characteristics*					
Size (mm)*	15.7 ± 3.5	(7.0–20.0)	12.9 ± 5.9	(1.0–20.0)	< 0.001
Location (head/body–tail)	57/55		35/63		0.036
Histological tumor grade (well/moderately/poorly)	56/45/11		43/48/7		0.408
TNM stage (I/II/III/IV)	83/26/3/0		76/21/0/1		0.287

Except where indicated, the data are expressed as percentages, with the numerator/denominator in parentheses

* Descriptive statistics are expressed as mean ± standard deviation and ranges in parentheses

PDAC = pancreatic ductal adenocarcinoma; MRI = magnetic resonance imaging; CEA = carcinoembryonic antigen; CA19-9 = carbohydrate antigen 19-9

### MRI features of small PDACs

Interobserver agreements were substantial to almost perfect (κ values: 0.67–0.96) (Table 2). Tumors were well demarcated in 27.1% on T1-weighted imaging, 22.9% on T2-weighted imaging, and 42.4% on DWI among all PDACs, with the proportion being significantly higher on DWI than on T1-weighted imaging (*P* < 0.001) and T2-weighted imaging (*P* < 0.001), but not significantly different between T1- and T2-weighted imaging (*P* = 0.666) (Table 3). Abnormal tumor signal intensity was observed more frequently on T1-weighted imaging than on DWI (*P* = 0.027) and T2-weighted imaging (*P* < 0.001), and more frequently on DWI than on T2-weighted imaging (*P* < 0.001). Abnormal distal parenchymal signal intensity was more frequently observed on T1-weighted imaging than on DWI (*P* = 0.012) and T2-weighted imaging (*P* < 0.001), whereas no significant difference was observed between DWI and T2-weighted imaging (*P* = 0.537). In additional exploratory analyses, the distribution of well- and ill-demarcated tumors did not significantly differ according to T1-weighted imaging technique (2D vs. 3D, *P* = 0.154), T2-weighted imaging technique (SSFSE vs. FSE, *P* = 0.621), or DWI b-value (800 vs. 1000 s/mm², *P* = 0.676).

**Table 2:** Interobserver agreement for MRI features

Tumor demarcation	kappa (κ) value
	0.84 (0.76, 0.91)
*Tumor signal intensity*	
T1-weighted imaging	0.79 (0.66, 0.91)
T2-weighted imaging	0.74 (0.64, 0.83)
Diffusion weighted imaging	0.79 (0.68, 0.89)
*Distal parenchymal signal intensity*	
T1-weighted imaging	0.87 (0.80, 0.94)
T2-weighted imaging	0.67 (0.56, 0.77)
Diffusion-weighted imaging	0.85 (0.77, 0.92)
*Pancreatic parenchymal * ***atrophy***	
Focal atrophy	0.82 (0.70, 0.93)
Upstream parenchymal atrophy	0.93 (0.88, 0.98)
*Main pancreatic duct*	
Stenosis	0.94 (0.88, 1.00)
Dilatation	0.96 (0.92, 1.00)
*CBD stenosis with upstream dilatation*	0.95 (0.87, 1.00)
*Small retention cyst*	0.75 (0.66, 0.83)

Data in parentheses are 95% confidence intervals

MRI = magnetic resonance imaging; CBD = common bile duct

**Table 3:** Tumor delineation on MRI

All PDACs	MRI sequences					
(*n* = 210)	T1-weighted imaging		T2-weighted imaging		Diffusion weighted imaging	
*Tumor demarcation*						
Well-demarcated	27.1%	(57/210)	22.9%	(48/210)	42.4%	(89/210)
Ill-demarcated	72.9%	(153/210)	77.1%	(162/210)	57.6%	(121/210)
*Tumor signal intensity*						
Hypointensity	87.6%	(184/210)	1.4%	(3/210)	0.0%	(0/210)
Isointensity	12.4%	(26/210)	42.9%	(90/210)	19.5%	(41/210)
Hyperintensity	0.0%	(0/210)	55.7%	(117/210)	80.5%	(169/210)
*Distal parenchymal signal intensity*						
Hypointensity	60.5%	(127/210)	0.0%	(0/210)	0.0%	(0/210)
Isointensity	39.5%	(83/210)	67.1%	(141/210)	61.9%	(130/210)
Hyperintensity	0.0%	(0/210)	32.9%	(69/210)	38.1%	(80/210)

Data are expressed as percentages, with the numerator/denominator in parentheses

MRI = magnetic resonance imaging; PDAC = pancreatic ductal adenocarcinoma

### MRI features in well-demarcated PDACs (Fig. 2)

Among well-demarcated PDACs, DWI (79.5%, 89/112) showed a significantly higher rate of well-demarcated lesions than T1-weighted imaging (50.9%, 57/112; *P* < 0.001) or T2-weighted imaging (42.9%, 48/112; *P* < 0.001). Thirty-eight PDACs (33.9%) were well demarcated only on DWI, whereas 5 lesions (4.5%) that were ill demarcated on DWI were well demarcated on both T1- and T2-weighted imaging.

**Fig. 2:**
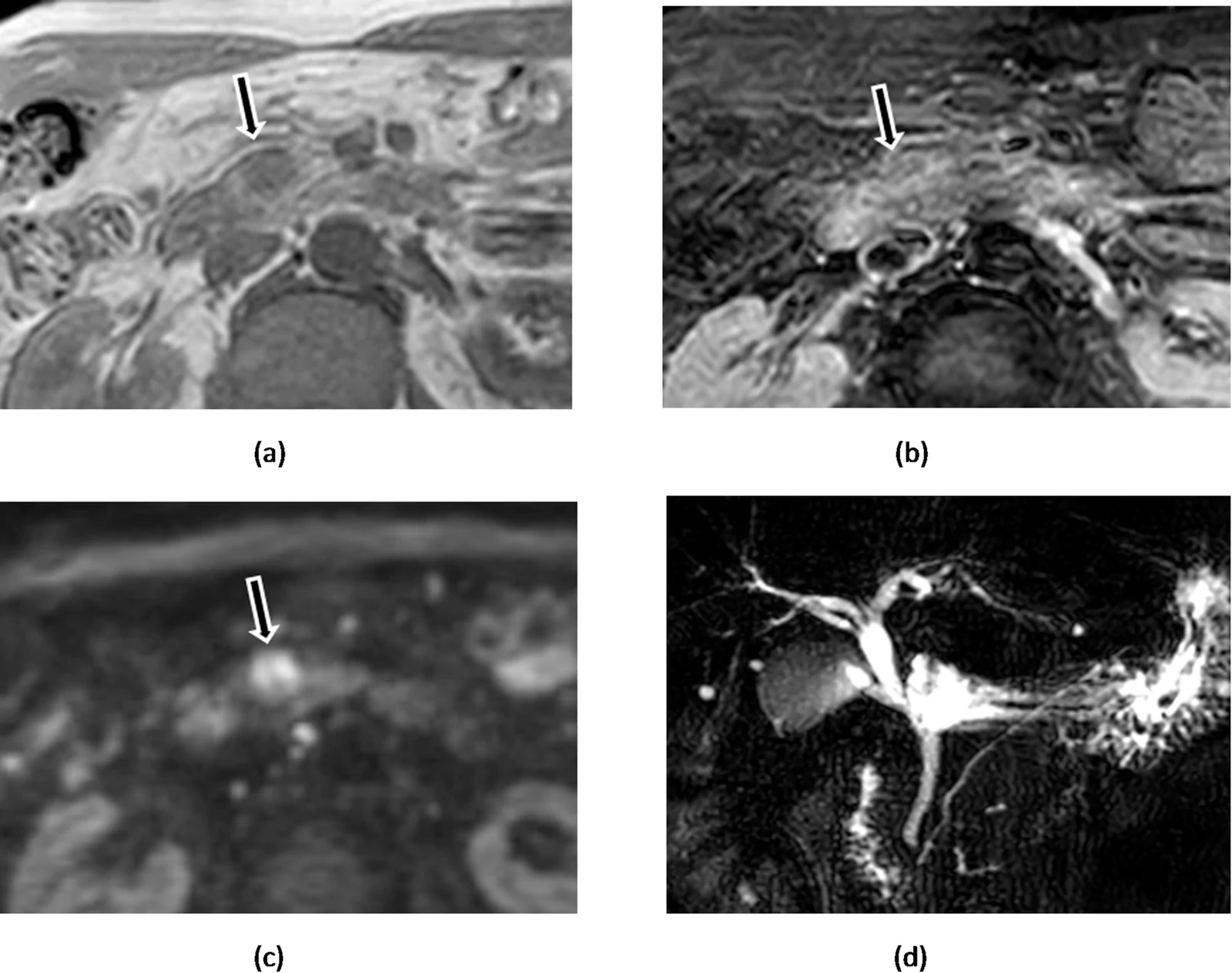
A 72-year-old male patient with pancreatic ductal adenocarcinoma in the pancreatic head measuring 14 mm. T1-weighted (**a**) and diffusion-weighted (**c**) images demonstrate a well-demarcated tumor (arrows) in the pancreatic head. The tumor is undetectable on fat-saturated T2-weighted imaging (**b**). No stenosis or dilatation of the main pancreatic duct distal to the tumor is observed on magnetic resonance cholangiopancreatography (**d**)

On T1-weighted imaging, 92.9% of tumors appeared hypointense (Table 4). On T2-weighted imaging, 1.8% appeared hypointense and 67.9% appeared hyperintense. On DWI, 92.9% appeared hyperintense. Abnormal tumor intensity was observed more frequently on T1-weighted imaging and DWI than on T2-weighted imaging (both *P* < 0.001). Abnormal distal parenchymal signal intensity was observed more frequently on T1-weighted imaging than on T2-weighted imaging (*P* = 0.006) or DWI (*P* < 0.001).

On T1-weighted imaging, focal and upstream parenchymal atrophy were detected in 12.5% and 32.1% of patients, respectively. On T2-weighted imaging and MRCP, MPD stenosis and dilatation were found in 69.6% and 61.6% of patients, respectively. CBD stenosis with upstream dilatation was noted in 15.2% of patients, while small retention cysts were observed in 55.4% of patients.

**Table 4:** Comparative analysis of well- and ill-demarcated PDACs

	Well-demarcated PDAC		Ill-demarcated PDAC		*P*-value
	(*n* = 112)		(*n* = 98)		
*Tumor signal intensity*					
T1-weighted imaging					0.020
Hypointensity	92.9%	(104/112)	81.6%	(80/98)	
Isointensity	7.1%	(8/112)	18.4%	(18/98)	
Hyperintensity	0.0%	(0/112)	0.0%	(0/98)	
T2-weighted imaging					< 0.001
Hypointensity	1.8%	(2/112)	1.0%	(1/98)	
Isointensity	30.4%	(34/112)	57.1%	(56/98)	
Hyperintensity	67.9%	(76/112)	41.8%	(41/98)	
Diffusion-weighted imaging					< 0.001
Hypointensity	0.0%	(0/112)	0.0%	(0/98)	
Isointensity	7.1%	(8/112)	33.7%	(33/98)	
Hyperintensity	92.9%	(104/112)	66.3%	(65/98)	
*Distal parenchymal signal intensity*					
T1-weighted imaging					< 0.001
Hypointensity	42.0%	(47/112)	81.6%	(80/98)	
Isointensity	58.0%	(65/112)	18.4%	(18/98)	
Hyperintensity	0.0%	(0/112)	0.0%	(0/98)	
T2-weighted imaging					0.012
Hypointensity	0.0%	(0/112)	0.0%	(0/98)	
Isointensity	75.0%	(84/112)	58.2%	(57/98)	
Hyperintensity	25.0%	(28/112)	41.8%	(41/98)	
Diffusion-weighted imaging					< 0.001
Hypointensity	0.0%	(0/112)	0.0%	(0/98)	
Isointensity	86.6%	(97/112)	33.7%	(33/98)	
Hyperintensity	13.4%	(15/112)	66.3%	(65/98)	
*Parenchymal atrophy*					
Focal atrophy	12.5%	(14/112)	15.3%	(15/98)	0.689
Upstream atrophy	32.1%	(36/112)	43.9%	(43/98)	0.088
*Main pancreatic duct*					
Stenosis	69.6%	(78/112)	93.9%	(92/98)	< 0.001
Dilatation	61.6%	(69/112)	90.8%	(89/98)	< 0.001
*CBD stenosis*	15.2%	(17/112)	5.1%	(5/98)	0.023
*Small retention cyst*	55.4%	(62/112)	57.1%	(56/98)	0.889

Data are expressed as percentages, with the numerator/denominator in parentheses

PDAC = pancreatic ductal adenocarcinoma; CBD = common bile duct

### ***MRI features in ill-demarcated PDACs*** (Table 4) (Fig. 3)

Abnormal tumor signal intensity was observed more frequently on T1-weighted imaging than on DWI (*P* = 0.001) and T2-weighted imaging (*P* < 0.001). Abnormal distal parenchymal signal intensity was observed more frequently on T1-weighted imaging than on DWI (*P* = 0.004) and even less commonly on T2-weighted imaging (*P* < 0.001). Focal and upstream parenchymal atrophy were observed in 15.3% and 43.9% of patients, respectively. MPD stenosis and dilatation were observed in 93.9% and 90.8% of patients, respectively. CBD stenosis with upstream dilatation was detected in 5.1% of patients, while small retention cysts were identified in 57.1% of patients.

**Fig. 3:**
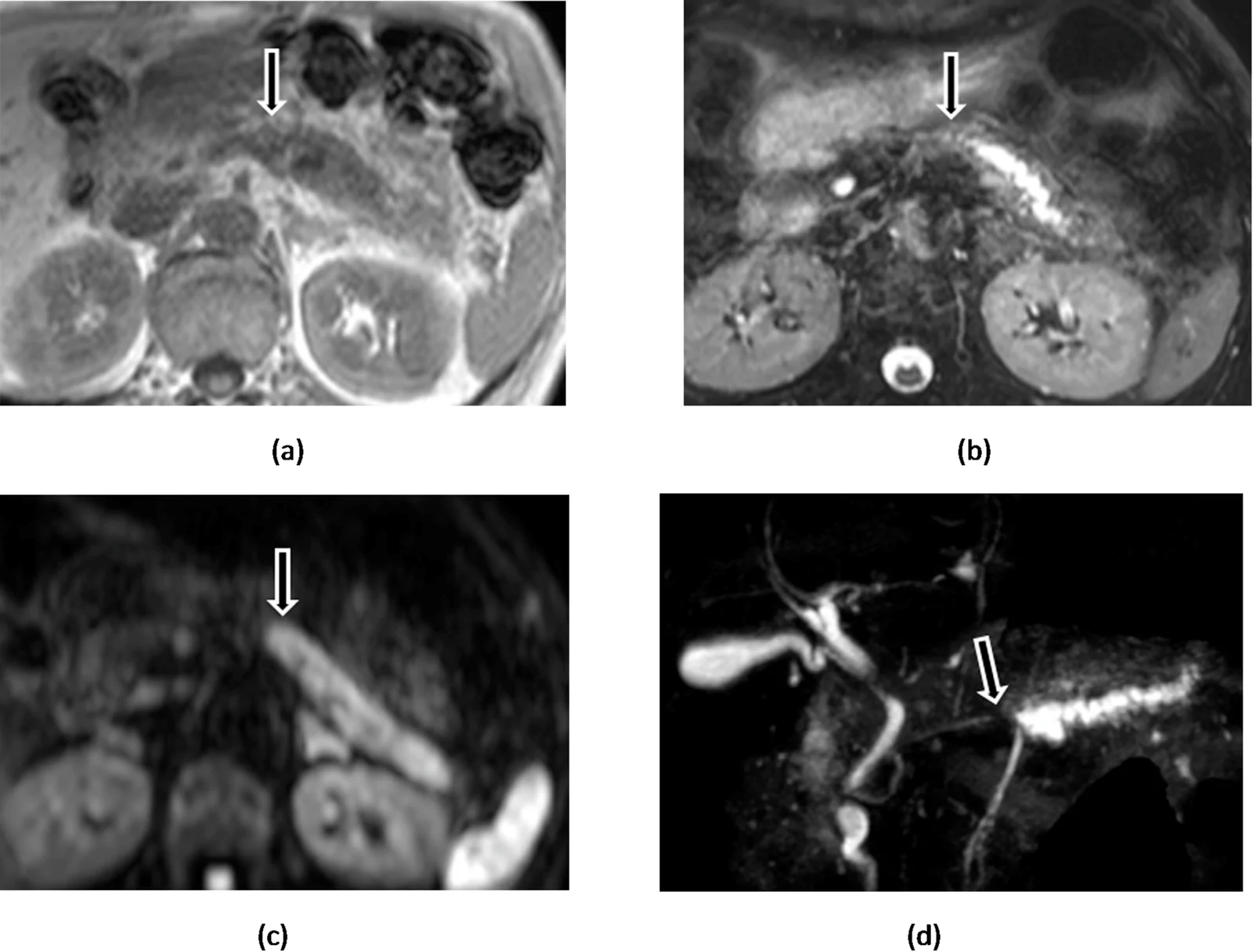
A 73-year-old female patient with pancreatic ductal adenocarcinoma in the pancreatic body measuring 10 mm. The tumor (arrow) is ill-demarcated, as indicated by hyperintense signal changes in the pancreatic tail on T1-weighted (**a**), fat-saturated T2-weighted (**b**), and diffusion-weighted (**c**) images. Stenosis of the main pancreatic duct with upstream ductal dilatation is clearly visible on magnetic resonance cholangiopancreatography (**d**)

### ***Comparison of well- with ill-demarcated PDACs*** (Table 4)

Abnormal tumor intensity was observed more frequently in well-demarcated tumors than in ill-demarcated ones on T1-weighted imaging (*P* = 0.020), T2-weighted imaging (*P* < 0.001), and DWI (*P* < 0.001).

Abnormal distal parenchymal signal intensity was observed more frequently in ill-demarcated tumors than in well-demarcated ones on T1-weighted imaging (*P* < 0.001), T2-weighted imaging (*P* = 0.012), and DWI (*P* < 0.001). The frequencies of MPD stenosis and dilatation were significantly higher in the ill-demarcated tumors (both *P* < 0.001). In contrast, CBD stenosis with upstream dilatation was observed more frequently in well-demarcated tumors than in ill-demarcated ones (*P* = 0.023). No significant differences were observed in the presence of focal (*P* = 0.689) or upstream parenchymal atrophy (*P* = 0.088), or small retention cysts (*P* = 0.889).

Ill-demarcated tumors were also more frequently associated with at least one secondary sign (99.0%, 97/98 vs. 84.8%, 98/112; *P* = 0.002).

### ***Independent factors associated with ill-demarcated PDACs*** (Table 5)

Tumor size (*P* = 0.002) and MPD dilatation (*P* = 0.002) were identified as independent factors associated with ill-demarcated small PDACs.

**Table 5:** Univariate and multivariate logistic regression analyses

	Well-demarcated PDAC	Ill-demarcated PDAC	Univariate analysis		Multivariate analysis	
	(*n* = 112)	(*n* = 98)	Odds ratio	*P*-value	Odds ratio*	*P*-value
*Sex (male/female)*	49/63	52/46	1.45 (0.84, 2.51)	0.178		
Age (years)*	71.3 ± 9.6	69.9 ± 8.7	0.98 (0.95, 1.01)	0.267		
*Opportunities for detecting PDAC*				0.299		
Incidental detection during evaluation of non-PDAC conditions	45.5%	53.6%	0.67 (0.30, 1.45)			
Medical check-up	27.3%	18.6%	1.16 (0.60, 2.22)			
Symptoms	27.3%	27.8%	1.0			
*Tumor markers*						
CEA (> 5.68 ng/mL)	13.5%	8.2%	0.58 (0.22, 1.39)	0.223		
CA19-9 (> 37.0 U/mL)	51.8%	28.9%	0.38 (0.21, 0.67)	< 0.001	0.57 (0.29, 1.13)	0.106
*Tumor characteristics*						
Size (mm)*	15.7 ± 3.5	12.9 ± 5.9	0.86 (0.80, 0.92)	< 0.001	0.88 (0.81, 0.95)	0.002
Location (head/body–tail)	57/55	35/63	0.54 (0.31, 0.93)	0.027	0.87 (0.44, 1.70)	0.682
Histological tumor grade (well/moderately/poorly)	56/45/11	43/48/7	1.09 (0.71, 1.67)	0.697		
TNM stage (I/II/III/IV)	83/26/3/0	76/21/0/1	0.85 (0.49, 1.46)	0.556		
*Parenchymal atrophy*						
Focal atrophy	12.5%	15.3%	1.27 (0.57, 2.80)	0.557		
Upstream atrophy	32.1%	43.9%	1.65 (0.94, 2.91)	0.080	1.09 (0.53, 2.21)	0.822
*Main pancreatic duct*						
Stenosis	69.6%	93.9%	6.68 (2.85, 18.43)	< 0.001	2.85 (0.95, 8.56)	0.056
Dilatation	61.6%	90.8%	7.01 (3.25, 16.96)	< 0.001	4.64 (1.73, 12.46)	0.002
*CBD stenosis*	15.2%	5.1%	0.30 (0.10, 0.79)	0.014	0.51 (0.15, 1.75)	0.276
*Small retention cyst*	55.4%	57.1%	1.08 (0.62, 1.86)	0.795		

Data in parentheses are 95% confidence intervals

*Descriptive statistics are expressed as mean ± standard deviation

PDAC = pancreatic ductal adenocarcinoma; CEA = carcinoembryonic antigen; CA19-9 = carbohydrate antigen 19 − 9; CBD = common bile duct

## Discussion

This multicenter study demonstrated that nearly half of small PDACs are visually ill-demarcated on unenhanced MRI, highlighting a critical challenge in early pancreatic cancer diagnosis. Ill-demarcated PDACs were strongly associated with various imaging features, such as abnormal distal parenchymal signal intensity and MPD dilatation. Conversely, clinical characteristics and serum tumor markers provided limited diagnostic value. These findings underscore the importance of careful image interpretation and recognition of secondary imaging characteristics to avoid missed or delayed diagnosis of small PDACs.

In this context, ill-demarcated PDACs on unenhanced MRI accounted for a markedly higher proportion of small PDACs (46.7%) than the reported prevalence of isoattenuating tumors on contrast-enhanced computed tomography (CT) (11%–17.5%) [17, 22–27]. Despite this high prevalence, no significant differences were observed in patient sex, age, or mode of tumor detection. Serum CA19-9 levels more frequently remained within the normal range in ill-demarcated tumors than in well-demarcated tumors, indicating the limited value of clinical parameters for detecting small PDACs. Although ill-demarcated PDACs tended to be smaller in size, which may partly account for the lower frequency of CA19-9 elevation, and were more commonly located in the pancreatic body and tail, no significant differences were found in histological tumor grade or TNM stage. Altogether, these findings suggest that an ill-demarcated appearance represents an early imaging manifestation of PDAC rather than an aggressive biological phenotype.

Previous studies have reported that DWI allows clear delineation in only approximately 39%–50.9% of PDACs, potentially owing to hyperintensity of the distal pancreatic parenchyma [9–11]. Similarly, we found that well-demarcated tumors comprised 42.4% of all small PDACs on DWI. In contrast, the delineation capability of T1- and T2-weighted imaging for PDACs has not been well-established. In our study, significantly more well-demarcated tumors were observed on DWI than on either T1- or T2-weighted imaging. Notably, a substantial proportion of ill-demarcated small PDACs remained inconspicuous, even on DWI, suggesting that reliance on lesion conspicuity alone, even with DWI, may be insufficient for early tumor detection.

Abnormal tumor signal intensity was observed more frequently on T1-weighted imaging than on DWI or T2-weighted imaging. However, abnormal distal parenchymal signal intensity was also more common on T1-weighted imaging, which may obscure tumor margins and reduce lesion conspicuity. The abnormal parenchymal signal changes observed on T1-weighted imaging may reflect tumor-associated pancreatitis and fibrosis, which reduce the intrinsic T1 signal of pancreatic parenchyma and diminish tumor-to-parenchyma contrast, as previously shown by Gabata et al. [13]. In both well- and ill-demarcated PDACs, abnormal distal parenchymal signal intensity was observed more frequently on T1-weighted imaging than on DWI or T2-weighted imaging. These findings indicate that when PDACs are not clearly delineated, careful assessment of pancreatic parenchymal signal abnormalities on T1-weighted imaging may provide a useful clue for tumor detection.

Previous studies have highlighted the significance of secondary signs, such as parenchymal atrophy, MPD stenosis and upstream dilatation, and small retention cysts adjacent to the tumor, in the diagnosis of isoattenuating PDACs on contrast-enhanced CT [14, 17, 23, 24, 27, 28]. Fukukura et al. [27] demonstrated that the frequency of secondary signs did not differ between visually hypoattenuating and isoattenuating PDACs on contrast-enhanced CT. However, data on secondary signs of PDAC on MRI remain limited [29, 30]. Moreover, to the best of our knowledge, differences in secondary signs on MRI between well-demarcated and ill-demarcated PDACs have not been investigated. In our study, ill-demarcated tumors were more frequently associated with at least one secondary sign than well-demarcated tumors, highlighting the importance of careful assessment of secondary signs in ill-demarcated PDACs.

MPD stenosis and dilatation were observed in 81.0% and 75.2% of all patients with PDAC, respectively. These frequencies were comparable to those reported by Kim et al. [29], who described MPD stenosis in 92.0% and MPD dilatation in 78.5% of patients. In our study, the frequencies of MPD stenosis and dilatation were significantly higher in ill-demarcated tumors. Furthermore, MPD dilatation was identified as an independent factor associated with ill-demarcated small PDAC. This relationship may help explain why ill-demarcated tumors were smaller and less frequently associated with elevated CA19-9 levels, yet more frequently accompanied by MPD abnormalities. A smaller tumor burden may contribute to lower CA19-9 levels, whereas MPD stenosis and dilatation may reflect the degree of ductal obstruction rather than tumor size alone. Even a small PDAC may induce marked secondary ductal and parenchymal changes when located close to the MPD [27]. These findings are supported by previous reports showing that tumor-associated pancreatitis secondary to MPD stenosis can induce abnormal distal parenchymal signal intensity, thereby impairing clear tumor delineation [9, 10]. Accordingly, our findings suggest that ill-demarcated PDACs may represent a less mass-forming and more duct-centered imaging pattern than well-demarcated tumors, although this hypothesis requires further pathological validation. From a diagnostic perspective, secondary signs on MRI, particularly MPD stenosis or dilatation on T2-weighted imaging and MRCP, should be considered not merely as ancillary findings but as important diagnostic clues for PDAC, especially when the tumor is not clearly delineated.

Pancreatic parenchymal atrophy was detected in 51.4% of all patients with PDAC, which is comparable to the 42.0% reported by Kim et al. [29]. Small retention cysts adjacent to the tumor were noted in 56.2% of all patients. Vullierme et al. [12] reported small retention cysts in 52.3% (34/65) of patients with pancreatic intraepithelial neoplasia (PanIN), suggesting that the presence of small retention cysts on MRI may be a significant predictor of PanIN. These findings raise the possibility that ill-demarcated PDACs share imaging features with PanIN. To our knowledge, no MRI-based comparative studies of PanIN and PDAC have been conducted, and this issue warrants further investigation.

More broadly, the specificity of these secondary MRI findings is limited. Findings such as MPD stenosis or dilatation, distal pancreatic parenchymal signal abnormality, parenchymal atrophy, and small retention cysts may also be observed in non-neoplastic conditions, including inflammatory change, chronic pancreatitis, or precursor lesions such as PanIN. Therefore, reliance on secondary signs alone may lead to false-positive interpretation in clinical practice. These findings should be interpreted in conjunction with direct tumor visibility and other imaging findings, rather than as stand-alone diagnostic markers of PDAC.

Our study has some limitations. First, the retrospective design may have produced selection bias. Second, detailed information on the specific non-PDAC conditions included in the category of incidental detection during evaluation of non-PDAC conditions was not systematically collected; therefore, residual heterogeneity in clinical context and selection bias cannot be fully excluded. Third, MRI acquisition parameters varied across institutions because of the multicenter retrospective design, which may have influenced lesion conspicuity and the assessment of secondary findings, particularly in very small PDACs. In exploratory subgroup analyses, the distribution of well- and ill-demarcated tumor groups did not significantly differ according to T1-weighted imaging technique (2D vs. 3D; *P* = 0.154), T2-weighted imaging technique (SSFSE vs. FSE; *P* = 0.621), or DWI (b = 800 vs. b = 1000 s/mm²; *P* = 0.676). Nevertheless, because slice thickness varied across multiple sequences and overlapped with sequence type and institutional protocols, stable stratified analysis for this parameter was difficult. Accordingly, the potential influence of residual technical heterogeneity cannot be fully excluded. Fourth, MRI features were assessed visually rather than quantitatively using objective metrics; however, interobserver agreement ranged from substantial to almost perfect (κ = 0.67–0.96), supporting the reproducibility of this classification. Finally, this study evaluated only unenhanced MRI. Although contrast-enhanced MRI can improve tumor conspicuity, non-contrast MRI sequences are more broadly applicable in surveillance or screening settings, where minimizing patient burden is desirable. Future studies incorporating contrast-enhanced MRI may further clarify the imaging characteristics of visually ill-demarcated small PDACs.

In conclusion, MRI fails to clearly delineate nearly half of small PDACs. The likelihood of an ill-demarcated appearance is associated with tumor size, abnormal distal parenchymal signal intensity, and the presence of MPD stenosis and dilatation. Abnormal distal parenchymal signal intensity is observed more frequently on T1-weighted imaging than on DWI and T2-weighted imaging. Most ill-demarcated small PDACs manifest with secondary signs, suggesting the importance of careful evaluation of these signs in their diagnosis.

## Electronic Supplementary Material

Below is the link to the electronic supplementary material.


Supplementary Material 1


## Data Availability

The data supporting the findings of this study are available from the corresponding author upon reasonable request due to privacy restrictions.

## Declarations

### Ethial approval

This multicenter retrospective study was approved by the institutional review boards of all participating institutions, and the requirement for informed consent was waived.
