# Supplementary material for: Characteristics of ill-demarcated small pancreatic ductal adenocarcinoma on MRI: a multi-institutional retrospective cohort study
Source: Jpn J Radiol. 2026 May 20;44(9):1648–59. doi: 10.1007/s11604-026-02003-8 (PMC13526109; doi:10.1007/s11604-026-02003-8)
Supplement: Supplementary file 1 [file 11604_2026_2003_MOESM1_ESM.docx]

**Supplementary Table 1A. MRI sequences and parameters for T1- and T2-weighted imaging**

|  | **2D T1-weighted imaging** | |  | **3D T1-weighted imaging** | |  | **SSFSE T2-weighted imaging** | |  | **FSE T2-weighted imaging** | |
| --- | --- | --- | --- | --- | --- | --- | --- | --- | --- | --- | --- |
|  | **1.5 T** | **3.0 T** |  | **1.5 T** | **3.0 T** |  | **1.5 T** | **3.0 T** |  | **1.5 T** | **3.0 T** |
|  | **(n = 84)** | **(n = 123)** |  | **(n = 71)** | **(n = 111)** |  | **(n = 83)** | **(n = 115)** |  | **(n = 76)** | **(n = 117)** |
| **Repetition time (ms)** | 130–180 | 130–300 |  | 3.0–5.0 | 3.1–5.0 |  | infinity | infinity |  | Respiratory | Respiratory |
|  |  |  |  |  |  |  |  |  |  | intervals | intervals |
| **Echo time (ms)** | 2.3–2.4 /  4.2–4.5 | 1.1–1.2 /  2.3–2.4 |  | 2.3–2.4 /  4.2–4.5 | 1.1–1.2 /  2.3–2.4 |  | 80–150 | 75–150 |  | 80–120 | 65–90 |
| **Flip angle (°)** | 60–90 | 60–80 |  | 10–16 | 8–15 |  | 90 | 90 |  | 90 | 90 |
| **Parallel acquisition** | 1–2.0 | 1–2.2 |  | 1–2.0 | 1–2.3 |  | 1–2.0 | 1–2.2 |  | 1–1.8 | 1–2.5 |
| **b-value (s/mm^2^)** | – | – |  | – | – |  | – | – |  | – | – |
| **Field of view (mm × mm)** | 262–400 x  330–400 | 300–400 x  340–400 |  | 240–400 x  350–400 | 240–420 x  340–420 |  | 246–400 x  320–400 | 320–380 x  350–380 |  | 230–360 x  350–360 | 250–400 x  320–400 |
| **Matrix (phase x frequency)** | 126–224 x  128–384 | 136–260 x  224–320 |  | 129–230 x  256–352 | 138–320 x  240–448 |  | 128–256 x  128–320 | 173–256 x  256–384 |  | 67–320 x  128–320 | 165–384 x  256–384 |
| **Slice thickness (mm)** | 4.0–7.0 | 4.0–8.0 |  | 3.0–5.0 | 1.5–5.0 |  | 4.0–8.0 | 4.0–8.0 |  | 4.0–6.0 | 4.0–8.0 |
| **Number of slices** | 18–48 | 24–50 |  | 30–108 | 24–133 |  | 18–48 | 24–35 |  | 20–48 | 20–45 |
| **Number of excitations** | 1 | 1 |  | 1 | 1 |  | 1 or 2 | 1 |  | 1–4 | 1 or 2 |
| **Number of breath-holds** | 1 | 1 |  | 1 | 1 |  | 1 | 1 |  | Free breathing | Free breathing |

Data are presented as overall ranges.

MRI = magnetic resonance imaging; SSFSE = single-shot fast spin-echo; FSE = fast spin-echo

**Supplementary Table 1B. MRI sequences and parameters for MRCP and DWI**

|  | **2D MRCP** | |  | **3D MRCP** | |  | **DWI** | | |
| --- | --- | --- | --- | --- | --- | --- | --- | --- | --- |
|  | **1.5 T** | **3.0 T** |  | **1.5 T** | **3.0 T** |  | **1.5 T** | **3.0 T** | |
|  | **(n = 45)** | **(n = 86)** |  | **(n = 58)** | **(n = 111)** |  | **(n = 84)** | **(n = 126)** | |
| **Repetition time (ms)** | infinity | infinity |  | Respiratory | Respiratory |  | Respiratory intervals or | | Respiratory intervals or |
|  |  |  |  | intervals | intervals |  | 1,800–10,000 | | 3,332–7,500 |
| **Echo time (ms)** | 95–1,100 | 144–987 |  | 93–800 | 65–700 |  | 60–84 | | 52–87 |
| **Flip angle (°)** | 90 | 90 |  | 90 | 90 |  | 90 | | 90 |
| **Parallel acquisition** | 1–2.0 | 1–2.0 |  | 1–2.0 | 1–5.5 |  | 1.25–2 | | 1.25–2.5 |
| **b-value (s/mm^2^)** | – | – |  | – | – |  | 800 or 1,000 | | 800 or 1,000 |
| **Field of view (mm × mm)** | 205–360 x  256–360 | 240–350 x  240–350 |  | 250–380 x  250–380 | 240–400 x  260–400 |  | 280–400 x  280–400 | | 260–400 x  350–400 |
| **Matrix (phase x frequency)** | 167–256 x  230–512 | 192–320 x  256–512 |  | 179–306 x  224–320 | 182–400 x  192–406 |  | 56–160 x  110–192 | | 67–192 x  96–256 |
| **Slice thickness (mm)** | 5.0–50.0 | 3.0–50.0 |  | 0.9–2.5 | 1.0–2.5 |  | 4–8 | | 4–7 |
| **Number of slices** | 1–24 | 1–30 |  | 80–200 | 62–240 |  | 18–48 | | 24–50 |
| **Number of excitations** | 1 or 2 | 1 or 2 |  | 1–3 | 1–3 |  | 2–14 | | 1–10 |
| **Number of breath-holds** | 1 | 1 |  | Free breathing | Free breathing |  | Free breathing | | Free breathing |

Data are presented as overall ranges.

MRI = magnetic resonance imaging; MRCP = magnetic resonance cholangiopancreatography; DWI = diffusion-weighted imaging
